# Netting Gut Disease: Neutrophil Extracellular Trap in Intestinal Pathology

**DOI:** 10.1155/2021/5541222

**Published:** 2021-10-19

**Authors:** Kai Chen, Li-Hua Shao, Feng Wang, Xiao-Fei Shen, Xue-Feng Xia, Xing Kang, Peng Song, Meng Wang, Xiao-Feng Lu, Chao Wang, Qiong-Yuan Hu, Song Liu, Wen-Xian Guan

**Affiliations:** ^1^Department of Gastrointestinal Surgery, Nanjing Drum Tower Hospital, The Affiliated Hospital of Nanjing University Medical School, Nanjing, China; ^2^Medical School of Nanjing University, Nanjing, China

## Abstract

Many gut disease etiologies are attributed to the presence of robust inflammatory cell recruitment. The recruitment of neutrophils plays a vital role in inflammatory infiltration. Neutrophils have various antimicrobial effector mechanisms, including phagocytosis, oxidative burst, and degranulation. It is suggested that neutrophils could release neutrophil extracellular traps (NETs) to kill pathogens. However, recent evidence indicates that neutrophil infiltration within the gut is associated with disrupted local immunological microenvironment and impaired epithelial barrier. Growing evidence implies that NETs are involved in the progression of many diseases, including cancer, diabetes, thrombosis, and autoimmune disease. Increased NET formation was found in acute or chronic conditions, including infection, sterile inflammation, cancer, and ischemia/reperfusion injury (IRI). Here, we present a comprehensive review of recent advances in the understanding of NETs, focusing on their effects in gut disease. We also discuss NETs as a potential therapeutic target in gut disease.

## 1. Introduction

Neutrophils are the first immune cells recruited into the inflammatory sites. They can recognize, phagocytize, and kill pathogens by producing reactive oxygen species, releasing lytic enzymes, and inducing neutrophil extracellular traps (NETs), a process termed “NETosis” [1]. NETs are extracellular structures composed of DNA fibers, chromatin, and granule proteins [1]. NETs not only have bactericidal activity but also play a crucial role in noninfectious conditions, including cancer [2], diabetes [3], thrombosis [4], and autoimmune disease [5]. Recently, NETs were suggested to lead to pathological changes in various gut diseases, including inflammatory bowel disease (IBD) [6, 7], colorectal cancer (CRC) [8, 9], and intestinal ischemia/reperfusion injury (IRI) [10, 11]. However, the relationship between NET formation and gut mucosal barrier remains largely unknown. Here, we describe the latest findings regarding NETs associated with intestinal infection, inflammation, cancer, and IRI. We also discuss how NETs serve as a future therapeutic target in the gut. Targeting NET formation and directly degrading NET structure could be promising novel strategies for therapeutic interventions in gut disease [10, 11].

## 2. NET Formation

Neutrophils are derived from myeloid progenitor cells in the bone marrow and are recruited to inflammatory tissue through a classical leukocyte recruitment cascade [12]. Once arrive at the inflammatory site, neutrophils could be activated by various stimuli such as bacteria [13, 14], fungi [15], viruses [16], and platelets [17]. In addition to these physiological materials, nonphysiological small compounds including phorbol myristate acetate (PMA) and calcium ionophores (CaI) could also activate neutrophils and have been used for mechanistic studies [18]. Neutrophils exert many biological functions including chemotaxis, antimicrobial functions, phagocytosis, degranulation, and NET formation [12]. The first publication that described NETs demonstrated NETs are composed of web-like structures of DNA coated with histones, elastase, and myeloperoxidase (MPO) [1]. Under high-resolution scanning electron microscopy (SEM), NETs contain smooth stretches with a diameter of 15 to 17 nm and globular domains of around 25 nm [1].

To date, studies have shown that NETs are formed through two main pathways: lytic NET formation which depends on NADPH oxidase (NOX) and nonlytic NET formation independent of NOX. Lytic NET formation leads to neutrophil death, while the nonlytic pathway could occur without cell death [19, 20]. Fuchs et al. [19] reported that NETosis caused by *Staphylococcus aureus* (*S. aureus*) or PMA depended on reactive oxygen species (ROS) produced by NOX. After stimulation by PMA, the nuclei of neutrophils lost their shape, and the chromatin homogenized. The nuclear envelope and granular membranes then decomposed, allowing the NET components to mix. Finally, as the cell membrane broke, NETs were released [19]. The NOX-independent pathway could release NETs without plasma membrane disruption. After NET release, neutrophils are still alive and reserve the ability to phagocytose and chemotaxis [20, 21]. The NOX-independent pathway requires neutrophil Toll-like receptor (TLR) 4 activation. The ability of neutrophils to produce NETs was diminished when treated mice with anti-TLR4 antibody (Ab) or knockout TLR4 [22, 23]. Reactive oxygen species (ROS) appear to promote NET formation whether or not the procedure is mediated by NOX. NOX-independent NET release requires ROS produced in the mitochondria [24]. ROS triggers the dissociation of neutrophil elastase (NE) from a membrane-associated complex into the cytosol and activates its proteolytic activity in a MPO-dependent manner [25]. NE translocates to the nucleus and partially degrades specific histones. Subsequently, MPO synergizes with NE in driving chromatin decondensation [26, 27].

NOX-dependent and NOX-independent NET formations converge to common outcomes, including activation of protein-arginine deiminase type 4 (PAD4), histone modification, chromatin decondensation, and NET release [28]. Histone posttranslation modification (PTM) could regulate chromatin decondensation and subsequent NET formation. Histone citrullination is the driver of chromatin decondensation, generated by PAD4 catalyzed hypercitrullination in the histones [29]. At the beginning of NETosis, the positive charge of histones decreased when chemically modified by enzymes such as PAD4 or NE, thus reducing the counterforces that hold the negatively charged DNA/chromatin together [27, 29]. Another modification of histone is histone acetylation was also suggested to promote NETosis when induced upon stimulation in human neutrophils [30]. Following chromatin decondensation, nuclear and plasma membrane ruptured to extrude NETs in the lytic manner. However, nonlytic NET formation could release NETs via vesicles without cell membrane rupture. Neutrophils without nuclei but have intact cell membranes; namely, “cytoplasts”, retain phagocytosis function [31]. Additionally, it should be noted that NETosis can be driven not only by biochemical signaling but also by material properties. Neubert et al. [32] have found that NETosis is highly organized into three distinct phases with a clear no-return point, determined by the chromatin status. Entropic chromatin swelling is the major physical driving force for cell morphology change and nuclear and plasma membrane rupture.

In summary, the pathways of NET formation have been partially formulated. The mechanisms are implicated, and further studies focusing on NET formation are awaited.

## 3. NETs and Enterogenic Infections

Microorganisms like bacteria [13, 14] and parasites [33] in the human gut have been proved to stimulate NET formation. The first study that described NETs suggested that NETs have an antibacterial function through sequestering bacteria and delivering a high local concentration of antimicrobial molecules [1]. During infection, NETs could persist for several days and eventually be dismantled by plasma nuclease DNase I [34, 35]. In the gut, NET formation was demonstrated to be a crucial manner of neutrophils inducing innate immune. Previous studies showed that PAD4-dependent NET generation is indispensable for intestinal clearance of *Citrobacter rodentium* (*C. rodentium*) [36]. *C. rodentium* colonized the intestine more rapidly when PAD4 was inhibited [36]. Consistently, Chaaban et al. [37] found that NET inhibition increased mortality, inflammation, and bacterial translocation in the necrotizing enterocolitis (NEC) model, suggesting the importance of neutrophil-mediated NET formation in preventing systemic bacterial dissemination during NEC. Although NETs may be critical in combating specific infections, evidence has showed that dysregulated NET formation could induce pathologies that impair the intestine epithelium barrier. Marin-Esteban et al. [13] developed a coculture model of activated neutrophils with the enterocyte-like Caco-2 cells. The F-actin cytoskeletons of enterocyte-like cells were damaged in the presence of NETs. Crane et al. [38] suggested that NETs could assist enteropathogenic *Escherichia coli* (*E.coli*) and Shiga-toxigenic *E.coli* to remain attached to the intestinal mucosa via DNA strands. These results have implied that NETs may benefit pathogens in the gut more than hurt them under some specific circumstance.

## 4. NETs and Intestinal Injuries during Sepsis

Sepsis is a systemic disorder with a dysregulated host response caused by infection and is accompanied by multiple organ dysfunctions and a high risk of death [39]. Gut microbiota translocation is suggested as the driver of sepsis and organ injuries [40]. Intestinal barrier dysfunction can lead to bacterial translocation and the release of intestine-derived inflammatory factors, which enter the systemic circulation [41]. Although NETs may exert a protective function in early immune response in sepsis [42], increasing evidence shows that if dysregulated, NETs and the components could contribute to intestinal epithelium destruction during sepsis [43–45]. Abundant neutrophils were activated to release NETs in the gut in lipopolysaccharide- (LPS-) induced sepsis [45]. Elevated serum NETs are associated with intestinal injury in abdominal sepsis patients [44]. Sun et al. [44] suggested that NETs activated endoplasmic reticulum (ER) stress in the lethal septic shock model. TLR9 antagonist administration alleviated NET-induced damage in the intestinal epithelial cell monolayer through ER stress inhibition [44]. Collectively, these findings demonstrated that the release of NETs may lead to intestinal damage during sepsis.

## 5. NETs and IBD

IBD, including Crohn's disease (CD) and ulcerative colitis (UC), are characterized by aberrant immunological responses leading to chronic inflammation without tissue regeneration [46]. Recent studies show that elevated plasma NET levels are associated with IBD occurrence in patients and experimental models [6, 47–49]. NET presence has been demonstrated in biopsy samples from IBD patients [49]. Pentraxin (PTX) 3, stored in neutrophil granules, could be released in response to microbial recognition. Released PTX3 can partially localize in NETs [50]. Savchenko et al. [50] reported that the numbers of PTX3 were increased in the high histological granules of the inflammatory reaction in UC patients, indicating that NET release containing PTX3 may contribute to cell immune defense in inflamed colon tissue of UC patients.

However, in addition to making up a part of immune defense, NETs may serve as a detrimental factor in gut epithelial barrier function and lead to the pathogenesis of mucosal inflammation during IBD. Lin et al. [48] found that NETs could alter the integrity of tight junction and adherent junction proteins, inducing intestinal cell death. Consistently, NET treatment in UC lamina propria mononuclear cells could activate extracellular signal-regulated kinase (ERK) 1/2 pathway, thus enhancing the production of tumor necrosis factor-*α* (TNF-*α*) and interleukin-1*β* (IL-1*β*) [6]. Moreover, IBD is associated with a hypercoagulable state and thromboembolism [51]. NETs are suggested to induce hypercoagulable state and thromboembolic disorders. Degradation of NETs by DNase I could reverse coagulation time and reduce fibrin formation in the active UC group [52]. These data have concluded that NET formation during IBD could ultimately exacerbate mucosal inflammation and NET inhibition has a protective effect on this disorder.

## 6. NETs and Colorectal Cancer

Neutrophils make up a significant part of the inflammatory cell infiltrate in many models of cancer. These neutrophils infiltrated in the tumor microenvironment, leading to pro- or antitumorigenic functions. These pro- or antitumor effects depend on the type of neutrophils [53]. Specific tumor-mediated signals, such as transforming growth factor-*β* (TGF-*β*), are believed to induce the formation of a tumorigenesis (N2) phenotype. Neutrophils also show an antitumorigenic (N1) phenotype [53]. Similarly, NETs play a vital role in both inhibition and promotion of cancer progression. Arelaki et al. [54] suggested that NET structure could inhibit growth and induce apoptosis in colon cancer cells *in vitro*. However, increasing evidence indicates that excessive NET production in tumor microenvironment may facilitate tumor growth, invasion, and metastasis [8, 55, 56].

CRC is the world's fourth most deadly cancer with almost 900,000 deaths annually [57]. Liver is the most frequent site of CRC metastasis, as most intestinal mesenteric drainage enters through the hepatic portal venous system [57]. Despite early detection and treatment, metastases including lymphatic and distant metastases remain the leading cause of death in CRC patients [58]. Patients with CRC are exposed to increasing risk of venous thrombosis, accompanied with high procoagulant state [59]. Recently, considerable evidence has indicated that NETs are involved in CRC progression and metastatic dissemination, both in animal models and CRC patients. High numbers of blood and intratumor neutrophils in various solid tumors were reported to predict poor clinical outcome [55, 60]. NET levels increased in the circulation of CRC patients compared with healthy volunteers. In addition, enhanced NET production was associated with postoperative complications such as longer hospitalization and increased mortality [8]. In this part, the role of NETs as a detrimental factor in cancer progression was discussed, especially as it relates to CRC, in terms of tumor growth, tumor-associated thrombosis, and liver metastasis.

### 6.1. NET Production in Tumor Microenvironment

**Figure 1:**
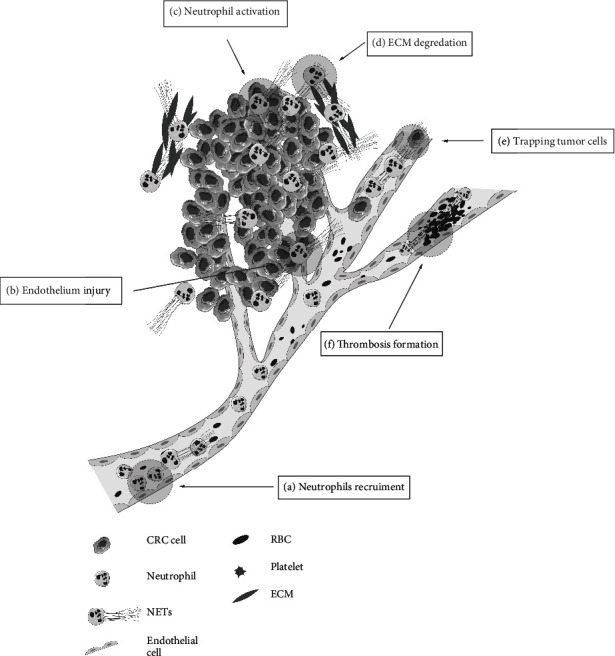
The role of neutrophil extracellular traps (NETs) in colorectal cancer (CRC) tumor microenvironment. (a) Neutrophils are recruited to the primary tumor by granulocyte colony-stimulating factor (G-CSF) released by tumor cells. (b) NETs could damage endothelial and increase the permeability of endothelium. (c) Tumor cells release interleukin-8 (IL-8) and exosomes, activating surrounding neutrophils to generate NETs. (d) Neutrophil elastase (NE) and matrix metalloproteinase-9 (MMP-9) on NETs degrade the laminin of extracellular matrix (ECM). The remodeled laminin could activate the *α*3*β*1 signaling pathway, inducing the proliferation of dormant cancer cells. (e) Tumor cells are sequestered by DNA webs of NETs, facilitating hematogenous metastasis. (f) NETs serve as a physical scaffold for thrombus growth by binding platelets and red blood cells (RBCs).

Initially, systemic infection was considered necessary to induce NET formation in cancer. Minor or severe systemic infections in tumor-bearing mice could activate neutrophils and induce NET release [56]. Growing evidence has yet suggested that recruitment of neutrophils and formation of NETs play a crucial role in tumor microenvironment. In various solid tumors, including CRC, the presence of NETs was detected within the tumor microenvironment [61, 62] (Figure 1). Release of the granulocyte colony-stimulating factor (G-CSF) into the bloodstream assists tumors in recruiting neutrophils for NET formation [63]. Cell-free DNA (cf-DNA) derived from cancer cells can activate TLR9 signaling and promote IL-8 secretion in CRC [64]. Through secreting IL-8, cancer cells promoted the release of NETs. Alfaro et al. [55] found that IL-8 derived from tumors contributed to the chemotactic recruitment of granulocytic myeloid-derived suppressor cells (GrMDSC) and induced the formation of NETs in GrMDSC. Moreover, cancer cells could also secrete exosomes to regulate tissue microenvironment. Exosomes derived from cancer cells including CRC cells triggered IL-8 production and stimulated NETosis in neutrophils [60, 65].

Recruitment of neutrophils in tumor microenvironment promotes NET formation and facilitates tumor growth [62]. Compared with healthy volunteers, neutrophils from CRC patients could produce more NETs *in vitro* [8]. Furthermore, recent studies have suggested that NET-associated proteases play a crucial role in the spread of cancer [66]. Albrengues et al. [66] revealed that two NET-associated proteases, namely, neutrophil elastase (NE) and matrix metalloproteinase-9 (MMP-9), sequentially cleaved laminin of extracellular matrix (ECM) and promoted ECM degradation. The proteolytically remodeled laminin led to integrin *α*3*β*1 signaling activation in cancer cells, inducing the proliferation of dormant cancer cells [66].

### 6.2. NETs Promote the High Procoagulant Status of CRC

CRC patients face a higher risk of venous thrombosis due to a state of high coagulation. Markers of extracellular DNA traps were detected in the thrombus [67]. An increase of NETs was closely associated with cancer-associated thrombosis, procoagulant status, and blood clot formation [17, 59, 68].

Platelet-neutrophil interactions have been key initiators of NET release [17, 68]. P-selectin on activated platelets can induce platelet-mediated NETosis by binding to P-selectin glycoprotein ligand-1 (PSGL-1) on neutrophils [69]. Moreover, platelet-derived high-mobility group box 1 (HMGB1) can induce NETosis through neutrophil TLR4 activation [70]. Zhang et al. [71] reported that platelets from CRC patients could stimulate healthy neutrophils to extrude NETs, which could be inhibited by the depletion of HMGB1.

Platelets initiate the production of NETs. The latter, in return, triggers strong activation of platelets. The reciprocal action sets up a positive feedback loop. NETs provide a physical scaffold for thrombus growth by binding platelets, tumor-derived exosomes, and red blood cells (RBCs) [65, 67]. NETs could recruit RBCs, promote fibrin deposition, and induce red thrombus formation [67]. DNase or the anticoagulant heparin that dismantles the NET scaffold could prevent thrombus formation [65]. Additionally, NETs from CRC patients are more potent to activate platelets by inducing the exposure of phosphatidylserine (PS) on platelets, eventually leading to significantly enhanced procoagulant activity (PCA) [71]. These data suggest that NETs may serve as new therapeutic targets to reverse the thrombotic consequences of CRC.

### 6.3. NETs in the Metastasis of Colorectal Cancer

In the early stage of cancer progression, neutrophils can accumulate in premetastatic organs in response to factors released by cancer cells [61]. Previous studies indicated that NETs played an important role in cancer metastasis. Several studies in mice and humans have shown that high expression of NETs facilitated cancer metastasis in the liver, lung, and lymph nodes [72, 73]. Initially, NET deposition was observed in organ microvasculature as response to surgical stress or systemic infection in cancers. Tohme et al. [73] reported that NET formation was demonstrated occurred after major liver resection in metastatic CRC patients. The NET biomarker, circulating MPO-DNA, was associated with early metastatic recurrence in colon cancer patients [73]. In a cecal ligation and puncture (CLP) model, Cools-Lartigue et al. [72] demonstrated the microvascular NET deposition in the hepatic sinusoidal spaces. Lung carcinoma cells within DNA webs were associated with increased formation of hepatic micrometastases [72]. Recently, studies have suggested that tumor could drive NET deposition in end organs, with or without surgical stress or major infection [74]. Intravascular NETs display effects on increasing vascular permeability and promoting cancer cell extravasation [75]. In this sense, NETs serve to create a “premetastatic niche”.

**Figure 2:**
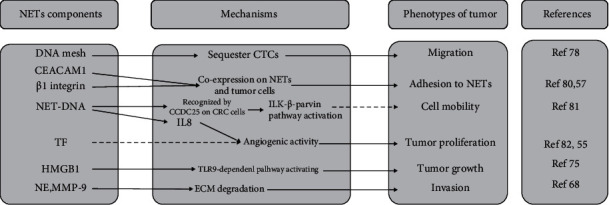
A simplified schematic representation summarizing the prometastasis effect of neutrophil extracellular traps (NETs) that have been described. The components of NETs, the mechanisms, and the phenotypes of tumor are listed. The black solid arrows represent links that have been demonstrated especially in colorectal cancer (CRC). Dotted lines represent links that were found in other solid tumors, whether could be applied to CRC requires further evidence. CEACAM1: carcinoembryonic antigen-related cell adhesion molecule 1; TF: tissue factor; HMGB1: high-mobility group box 1; NE: neutrophil elastase; MMP-9: matrix metalloproteinase-9; CTC: circulating cancer cell; ILK: integrin-linked kinase; TLR: Toll-like receptor; ECM: extracellular matrix.

In the “niche”, circulating tumor cells (CTCs) could be sequestered by DNA web of NETs, then lodging in the end organ and tissue, establishing new tumors [76]. The DNA mesh of NETs could trap CTCs but cannot kill or injure these metastasizing cells. Once wrapped around tumor cells, NETs and NET-associated proteins would directly interact with tumor cell membrane. It has been demonstrated that carcinoembryonic antigen-related cell adhesion molecule 1 (CEACAM1), a cell adhesion molecule expressed on endothelial cells, could promote liver metastasis of CRC [77]. The most recent study confirmed that CEACAM1 is present on both murine and human NETs. Blocking CEACAM1 on human NETs or knocking it down in mice could decrease the adhesion and migration of tumor cells by more than 50% [78]. CCDC25, a transmembrane protein expressed on CRC cells, is another newly discovered molecule promoting CRC metastasis. This protein senses NET-DNA and then activates the integrin-linked kinase (ILK)-*β*-parvin pathway to enhance cell mobility [79]. Najmeh et al. [56] identified *β*1 integrin as an abundant constituent of NETs. Present both *in vitro* and *in vivo*, *β*1 integrins, expressed on both tumor cells and NETs, mediated the adhesion of cancer cells to NETs. In the mechanistic investigations *in vitro*, NETosis triggered the release of HMGB1 and activated TLR9 pathways in cancer cells, further promoting tumor progression [73]. Following the dissemination and adhesion, CTCs can proliferate to form stable metastatic foci. Neutrophils and NETs, assumed to be sources of tissue factor (TF), could lead to angiogenic activity and facilitate tumor proliferation [54]. Consistently, a previous study found extracellular DNA presented on the surface of cancer cells, increased IL8 production, and facilitated angiogenesis of cancers [80]. Collectively, migration of neutrophils to premetastatic niches and subsequent NET formation allows the entrapment of CTCs, which leads to the formation of metastatic implants (Figure 2).

## 7. NETs in Intestinal IRI

Ischemia/reperfusion injury is a clinical problem, especially when the injury is involved in the gastrointestinal tract. Intestinal IRI occurs following acute mesenteric ischemia, traumatic or septic shock, burns, and surgical procedures. It can lead to multiple organ failure (MOF) and high mortality in critically ill patients [81–83]. Neutrophils may contribute to IRI in the intestine by forming extracellular traps [10]. Researchers have reported that NET biomarker citrullinated H3 (citH3) was elevated in several organs following IRI, including the kidneys [84], brain [85], liver [86], and myocardial tissues [87]. It has been recognized that NETs may exert harmful effects on these organs when coagulation, inflammation, and cell death are triggered [84–87]. Boettcher et al. [88] found that DNase I treatment could reduce intestinal injury during IRI, indicating that NETs may contribute to the development and progression of intestinal IRI [88]. In this section, we provide an overview of studies on the role of NETs in intestinal IRI. Ascher et al. [89] quantified leukocyte adherence and NET formation in IRI mesenteric venules by intravital imaging. During IRI, TLR4 expression in neutrophils was elevated, responsible for elevated NET formation [10]. NETs exacerbated the intestinal inflammation after IRI and destroyed the cytoskeleton structure of gut epithelial, along with functional integrity of tight junctions [10]. It was demonstrated that DNase I treatment could ameliorate tissue injury, apoptosis, and oxidative stress in the intestine [88]. In a rat model of trauma/hemorrhagic shock, early intravenous tranexamic acid administration attenuated NET formation and prevented disruption of tight junction protein [90]. These data indicate that NETs play a detrimental role in the pathogenesis of intestinal barrier during intestinal IRI. Moreover, as Hayase et al. [91] reported, extracellular histone and NET accumulation exacerbate remote liver injury after intestinal IRI. Administration of recombinant thrombomodulin (rTM) neutralized extracellular histones as well as attenuated liver injury. Consequently, NETs may be evaluated as early predictors or therapeutic targets of intestinal IRI in clinical trials.

## 8. NETs as Future Therapeutic Strategies

NETs can be regarded as promising therapeutic targets to improve the clinical outcome in gut diseases. Various therapeutic agents targeting NETs are clinically administrated in some conditions and are expected to have a protective effect on gut diseases. Inhibitors of molecules interfering with NET formation have been tested. Activated protein C (APC), a serine protease with anti-inflammatory activities, was confirmed to inhibit NETosis, as a part of anti-inflammatory function [92]. In a nonhuman primate model of *E.coli*-induced sepsis, pretreatment with APC abrogated release of MPO from neutrophils, an enzyme essential for NETosis [92]. Given that the enzyme PAD4 plays an important role in NET formation, it may be considered a potential therapeutic target [92–94]. Through inhibiting PAD4, NET release could be markedly reduced. In addition to intervening this enzyme, metabolic intermediates of NETosis could also serve as a therapeutic target. Deng et al. [93] developed a novel monoclonal antibody targeted citH3 generated by PAD2 and PAD4. Following blocked circulating citH3 and reduced NET formation, this antibody attenuated inflammatory responses and ameliorated acute lung injury (ALI). Recombinant thrombomodulin, a novel agent used for the treatment of patients with disseminated intravascular coagulation (DIC) in Japan [95], also displayed an effect on inhibiting NET formation *in vitro* [96, 97]. Hayase et al. [98] suggested rTM could attenuate liver injury by suppressing hepatic NET accumulation after intestinal IRI, thus improving survival. However, the mechanisms of the rTM-mediated NETosis inhibition are not clear and remain to be determined. It is possible that rTM exerts an inhibitory effect against TLR4-mediated signaling or bind to histones [96, 97].

In addition to interfering with NETosis, direct degradation of NETs is an alternative method. DNase I is an endonuclease that selectively cleaves the phosphodiester bond in DNA, the major structural component of NETs [1]. Intravenous administration of DNase I in the colitis mouse model can restore the mucosal barrier integrity and attenuate intestinal inflammation [48]. Xia et al. [99] developed a practical and clinically applicable delivery system providing long-term expression of DNase I: Human DNase I cDNA was put under the control of a liver-specific promoter, cloned into an adeno-associated virus (AAV) expression cassette. AAV-mediated DNase I reduced NET formation in CRC liver metastases. Moreover, heparin possessed the highest negative charge density of any biological macromolecule. Thus, it could strip positively charged histones from the DNA backbone of NETs to destabilize them [94, 100]. Found that the administration of heparin could restore pathological changes of ocular graft-vs.-host disease (oGVHD) dry eye induced by NETs. NETs represent a good target for DNase therapy. However, DNase I or heparin does not specifically target NETs but degrades extracellular DNA of any source. As a result, future studies of NET-specific therapies are required.

Previous studies have proved that NETs display a protective effect against infection, and NET inhibition attenuated anti-infection effects of neutrophils. Data implicating the degree to which NETs either inhibit or exacerbate the inflammation progression are controversial. In order to investigate the role of NETs in gut bacteria clearance, Saha et al. [36] challenged PAD4-deficient (PAD4^−/−^) mice and wild-type (WT) littermates with *C. rodentium*. They found luminal colonization of *C. rodentium* in PAD4^−/−^ mice unable to form NETs peaked between 11 and 14 days after infection, whereas WT mice suppressed the infection by 14 days. Moreover, an experiment was conducted to examine the outcome of NET inhibition in NEC model induced by *Klebsiella pneumoniae* infection [37]. Chloramine treatment inhibited NET formation and increased systemic inflammation, bacterial load, organ injury, and mortality in murine NEC [37]. In this sense, inhibition of NETs impaired the capacity of neutrophils defending enterogenic infection.

Although the strikingly different outcomes of NET inhibition in these studies may be derived from distinct animal models and stimuli, they bring contradiction to treatments targeting at NETs. Instead of complete depletion of NETs, new therapies should be developed which preserve the protective function against infection while preventing the excessive inflammation caused by NETs. Interestingly, Van Avondt et al. [101] found a solution through modifying signal inhibitory receptor on leukocyte-1 (SIRL-1). SIRL-1 intervention suppressed NET formation in response to *S. aureus* stimulation and preserved intracellular antimicrobial defense and ROS generation [101]. The findings provide the possibility to develop some new treating strategies, both attenuating the detrimental effect and retaining the protective effect of NETs.

## 9. Conclusion

NET-related researches have been shifted from innate immune defense to noninfectious diseases ranging from autoimmune disease to cancer. It is suggested that NETs aggravate inflammation, damage surrounding tissue, promote thrombosis, and facilitate cancer progression. It is essential to understand the role of NETs in gut disease, as neutrophil accumulation and activation are critical mechanisms of pathogenesis in the gut. As we presented in this review, NETs could affect the initiation and progression of IBD, CRC, and intestinal IRI. However, much remains unclear about the specific mechanism of NETs in intestine pathogenesis. Given the multitude of NET compositions, novel NET functions in unknown circumstances in the gut are likely to emerge in the future. Besides, it is not clear whether treatment agents like DNase and PAD4 inhibitors have side effects when targeting some receptors in addition to NETs. Therefore, in order to find the most precise molecule candidates for therapeutic targeting, a better understanding of the mechanisms of NETs in gut disease is needed. Moreover, NETs either present protective functions such as antimicrobial or pose harmful effects. How to balance the beneficial and detrimental effects of NETs would be a key point during novel drug development.

## Data Availability

All data generated or analyzed in this study are available from the corresponding authors on reasonable request.
